# Inferring pathogen-host interactions between *Leptospira interrogans* and *Homo sapiens* using network theory

**DOI:** 10.1038/s41598-018-38329-1

**Published:** 2019-02-05

**Authors:** Swapnil Kumar, Kumari Snehkant Lata, Priyanka Sharma, Shivarudrappa B. Bhairappanavar, Subhash Soni, Jayashankar Das

**Affiliations:** Gujarat Biotechnology Research Centre, Department of Science & Technology, Government of Gujarat, Gandhinagar, 382011 India

**Keywords:** Network topology, Regulatory networks

## Abstract

Leptospirosis is the most emerging zoonotic disease of epidemic potential caused by pathogenic species of *Leptospira*. The bacterium invades the host system and causes the disease by interacting with the host proteins. Analyzing these pathogen-host protein interactions (PHPIs) may provide deeper insight into the disease pathogenesis. For this analysis, inter-species as well as intra-species protein interactions networks of *Leptospira interrogans* and human were constructed and investigated. The topological analyses of these networks showed lesser connectivity in inter-species network than intra-species, indicating the perturbed nature of the inter-species network. Hence, it can be one of the reasons behind the disease development. A total of 35 out of 586 PHPIs were identified as key interactions based on their sub-cellular localization. Two outer membrane proteins (GpsA and MetXA) and two periplasmic proteins (Flab and GlyA) participating in PHPIs were found conserved in all pathogenic, intermediate and saprophytic spp. of *Leptospira*. Furthermore, the bacterial membrane proteins involved in PHPIs were found playing major roles in disruption of the immune systems and metabolic processes within host and thereby causing infectious disease. Thus, the present results signify that the membrane proteins participating in such interactions hold potential to serve as effective immunotherapeutic candidates for vaccine development.

## Introduction

Leptospirosis is one of the most common, dreaded and emerging zoonotic disease in human as well as cattle worldwide^1,2^. Weil’s disease or Weil’s syndrome, caused by pathogenic spp. of *Leptospira*, is an acute form of human leptospirosis and was first reported by Adolf Weil in 1886^3^. Humans may get infected through direct contact with urine, blood, or tissues of infected animals or indirect contact with contaminated mud or water. The acute form of leptospirosis is characterized by multi-organ complications such as cardiovascular collapse, acute renal and hepatic failure, meningitis, pneumonitis and pulmonary haemorrhage, which in turn can cause death^2^. It has been estimated to cause 1.03 million cases resulting in 58,900 deaths of humans per year^4^. These estimates have placed this disease as the most prominent zoonotic cause of morbidity and mortality in the world. Still, this data is believed to be underrating the disease burden, as the patients are usually misdiagnosed due to the resemblance of its symptoms with several other diseases like meningitis, yellow fever, kidney damage and hepatic failure^5–8^, which does not allow its proper treatment. Moreover, the lack of early diagnosis for effective medication and vaccination makes it difficult to control and treat the infection on time^2,9^. Therefore, there is an urgent need for development of novel and effective diagnostics and vaccine to control and prevent this emerging disease across the globe.

The pathogenic spp. of *Leptospira* comprises more than 250 distinct serovars as per their antigenic makeup^10,11^. In spite of the heterogeneity in morphology among different serovars of same species, they encompass similar genetic makeup and demonstrate similar virulence mechanism and pathogenesis^12^. The current information on mechanism of leptospiral pathogenesis and virulence is limited despite of numerous *in vitro* studies and advances made therein to understand pathophysiology of *L. interrogans*^13,14^. Albeit, it is indispensable to study *L. interrogans*-human protein interactions for pinning down the mechanism of pathogenesis used by the pathogen to cause the disease.

Interacting proteins involved in pathogen-host protein interactions (PHPIs) can be detected either experimentally or computationally. There are two key experimental approaches for the detection of interacting proteins: (i) binary approaches–like yeast two-hybrid (Y2H) and luminescence-based mammalian interactome mapping, and (ii) co-complex approaches–like co-immunoprecipitation (CoIP) combined with mass spectrometry (MS)^15–17^. But, these methods are expensive as well as time-consuming. Hence, there are many computational approaches which have been developed and applied to identify interacting proteins in high-throughput manner with better accuracy, coverage and efficiency^18,19^. These are mainly based on genomic features, protein sequences and structural information related to functional and interactional relationships^18,20–22^, including gene clustering methods^23,24^ and interologs based method^25–27^. Interologs are referred to those homologs which preserve their interaction ability: if two proteins interact with each other, their orthologs also have a tendency to interact with each other^28^. This method is not only applied to predict protein-protein interactions (PPIs) within an organism^29,30^, but also PPIs between pathogen and its host^31–33^.

With the dawn of “omics” technologies such as high throughput gene expression, genomic, transcriptomic and proteomic, a vast amount of biological data has been produced, which has shifted the focus of systems biology towards understanding the disease model through network biology approach^34–36^, especially where the PHPIs occur and affect various molecular functions and specialized biological actions of these proteins^27,37,38^. The availability of complete genome data of both the pathogen (*L. interrogans*)^39^ and the host (*H. sapiens*)^40^ act as requisite data source for the prediction of these PHPIs. Thus, *in silico* predictions of biologically meaningful PHPIs between *L. interrogans* and human will help to identify key proteins of *Leptospira* and their targets in human for further experimental investigations on their biological relevance.

In the present study, we have described the combined approach of *in silico* algorithms, network theory and functional annotations to explore, analyze and understand the *Leptospira*-human protein interactions. For this, first the inter-species protein-protein interactions between *L. interrogans* and *H. sapiens* were predicted, followed by the intra-species protein-protein interactions among proteins of *Leptospira* and also human. Subsequently, a protein interaction network between pathogen and host was constructed by mapping both the inter- and intra-species protein interactions. By detailed screening and analyses of PHPIs network, we were able to identify a set of key interactions involving bacterial membrane proteins (outer as well as inner) targeting human proteins. The structural analysis and functional annotation of interactors participating in PHPIs revealed their key structural features and helped to identify functions, processes and pathways related to bacterial pathogenesis.

## Results

### PHPIs map, statistical validation and quality assessment

A total of 586 pathogen-host protein interactions (PHPIs) among 638 proteins including 145 of *Leptospira* and 493 of human were identified as discussed in the methods section.

For determining the importance of the network organization in the PHPIs network, the Kolmogorov-Smirnov (KS) test was applied to calculate the *p*-value by comparing degree distributions between the random networks and the original one as discussed in the methods section. Thus, the *p*-value (0.003864818) obtained was statistically significant i.e., less than 0.5. This value showed that the hub-proteins and their sub-networks present in the network are not by chance.

Further, these interactions may contain a considerable amount of false positives. Hence for assessing the quality of interactions, sensitivity and specificity were calculated. True negative set of data is usually used for the calculation of parameters like sensitivity and specificity^41^. In our study, the Negatome v.2.0 database^42^ was used as a source of true negative set of interactions. A total of 6532 non-interacting proteins pairs from the database were processed for predicting interactions between them as discussed in the methods section. Out of 6532, total 32 pairs were predicted to have interactions between them. We calculated specificity as the percentage of true negatives predicted correctly out of 6532 non-interacting pairs. Thus, the specificity as calculated was 99.5% ((6532-32)/6532). Since, experimentally verified *Leptospira*-human protein-protein interactions (PPIs) are not available easily, we used previously reported data for comparison with our predicted data to assess the accuracy and sensitivity. Our predicted data was found to have 25 proteins of *L. interrogans* serogroup Icterohaemorrhagiae serovar Copenhageni (strain Fiocruz L1-130) (LIC) which were previously reported for playing an important role in the survival of bacteria and also responsible for infection in human^43^.

### Structural properties of networks

Biological networks across different species share their structural properties^36,44^. In our study also, all the inter-species as well as intra-species networks demonstrated similar pattern of properties like degree, degree distribution, clustering coefficient, betweenness and eigenvalue centrality properties. In spite of the common structural characteristics of these networks, functional and biophysical co-ordination is altered especially in case of inter-species network.

**Figure 1:**
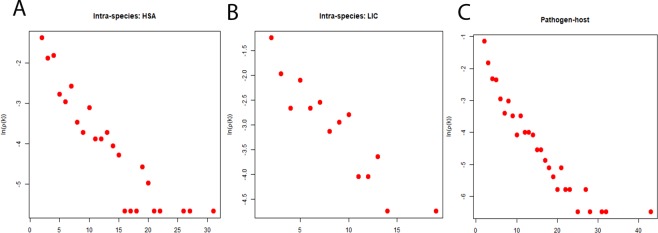
Degree distribution for the inter- and intra-species networks. Networks (**A**) HSA, (**B**) LIC and (**C**) PHPIs show that the degree distributions follow power law.

**Figure 2:**
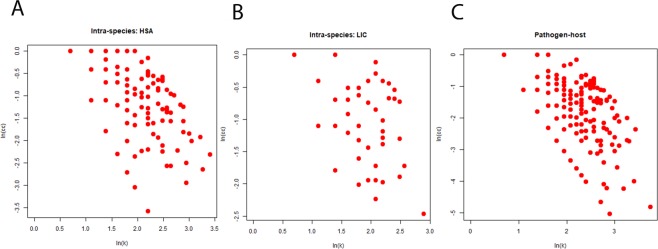
Degree-Clustering coefficient correlations for the inter- and intra-species networks. Networks (**A**) HSA, (**B**) LIC and (**C**) PHPIs show that the degree-clustering coefficient correlations are negatively correlated.

**Figure 3:**
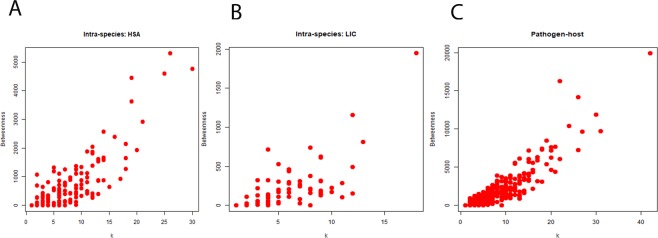
Degree-Betweenness correlations for the inter- and intra-species networks. Networks (**A**) HSA, (**B**) LIC and (**C**) PHPIs show that the degree-betweenness correlations are positively correlated.

**Figure 4:**
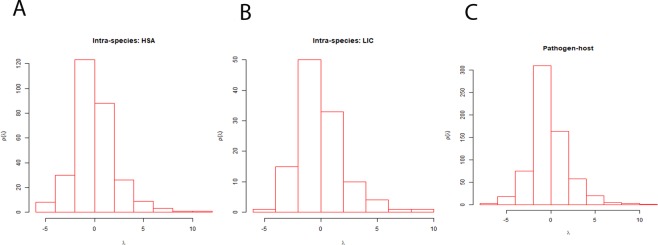
Eigen value distribution of inter- and intra-species networks. The plots depict similar distribution for all the three inter-species networks: (**A**) HSA; (**B**) LIC; and (**C**) PHPIs with a high degeneracy at zero.

Different structural properties of the networks have been summarized in the Table 1. The degree distribution of both inter-species and intra-species networks follow the property of power law (Fig. 1A–C) and scale free nature, which indicates the presence of nodes having very high degree in the network. These high degree nodes are known for keeping these networks robust towards external perturbations and found functionally important in various pathways^45^. The degree and clustering coefficient (CC) of both inter-species and intra-species networks are negatively correlated (Fig. 2A–C), as in case of many biological networks^46^. The value of average CC of the inter-species network was less than that of intra-species networks (Table 1). Regardless of exhibiting overall similar property in case of both the inter-species and intra-species networks, the differences which are crucial could be inferred from the clique structures analysis of these networks. The inter-species network exhibit less number of nodes having CC = 1 than the intra-species one as represented in Table 1. The CC values being one for nodes advocated complete sub-graph or clique formation in the network comprising of those nodes. The lower value of average CC indicates the presence of low number of cliques in a network^47^. Cliques are networks’ building blocks and make the underlying system highly stable and robust^48,49^. The inter-species network having less number of cliques as well as nodes with CC = 1 as compared to the intra-species network indicated that there was a disturbance in building blocks of the inter-species network and hence, causing instability in host. Thus, this could be one of the underlying reasons behind the development of disease. The importance of cliques can be understood in a better way after the functional exploration of hub proteins which are a part of these cliques. The analysis of inter-species network revealed not only importance of the hub proteins but also the structural patterns present in the network. The degree and betweenness of all three networks also exhibited similar pattern of correlation (Fig. 3A–C). This indicates that all three networks have similar pattern of organization within them. However, the inter-species network exhibits high values of average betweenness (<BC>) compared to the parental intra-species networks (Table 1). The eigenvalue distributions exhibit triangle like shape with long tail of distribution (Fig. 4A–C) connecting with the power law exponent of degree distribution as exhibited by various other biological networks^50,51^. Both the inter-species and intra-species networks exhibit high degeneracy at zero resembling the same as yielded by various other biological networks^50^.

**Table 1:** Network parameters for all inter-species and intra-species networks.

Network	N	NC	<k>	<CC>	N_CC_	<BC>
PHPI	655	1538	4.696183	0.1354224	4	964.2076
LIC	115	247	4.295652	0.4132618	10	150.2696
HSA	289	705	4.878893	0.3620147	28	379.8616

Total number of proteins (N) collected using database (described in the method section), Total number of connections (NC), Average degree (<k>), Average clustering coefficient (<CC>), Total number of nodes with CC = 1 in the network (${{\rm{N}}}_{{\rm{cc}}}$), and Average betweenness centrality (<BC>).

### Functionally important proteins

**Figure 5:**
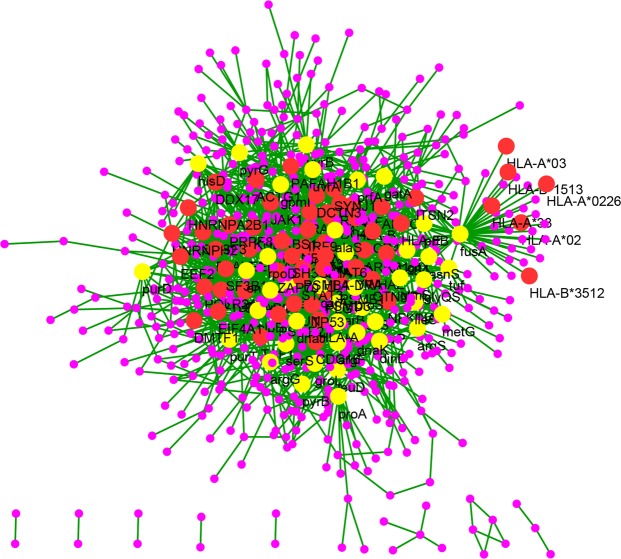
Hub proteins of *H. sapiens* and *L. interrogans* in PHPIs. Red colored nodes are hub proteins from *H. sapiens* and yellow colored nodes are hub proteins from *L. interrogans*.

After constructing the complete pathogen-host interactions network, all hub proteins of pathogen as well as of host were detected based on the degree of nodes as described in the methods section. The details of all hub proteins of both host and pathogen are summarized in Tables 2 and 3. The Ras-related C3 botulinum toxin substrate 1 (RAC1) protein had the highest degree value i.e. 31 among the host hub proteins (Table 2). The second highest degree protein of host was found to be TP53 which was interacting with 27 other proteins participating in the pathogen-host interactions (Fig. 5). Among hub proteins of pathogen (Table 3), the protein Elongation Factor G (EFG) coded by gene *fusA* of *L. interrogans* represented the highest degree value i.e. 42. Thus, the protein exhibits a very important role in the pathogen-host interactions. While, the protein exhibiting second highest degree is Chaperone protein ClpB (CLPB) which was interacting with 30 other proteins.

**Table 2:** Total 56 hub proteins of host *H. sapiens*.

Rank	Protein	Degree
1	RAC1	31
2	TP53	27
3	NFKB1, POLR2A	26
4	FYN	22
5	CDC42	21
6	ZAP70, SRSF3, HLA-DRA, JUN	20
7	DVL2	19
8	PRPF8, CTNNB1	18
9	B2M	17
10	SF3B1	16
11	EEF1A1, HNRNPH2, ARRB1	15
12	HDAC2, EEF2, SYNJ1, RAC2	14
13	CDC16, STAT3, HNRNPA2B1, HLA-A, PTBP1, ELAVL1, ACTG1, SNAPC4	13
14	HLA-B, HLA-E, PDIA3, JAK1, SH3GL1, ARPC3, CD4	12
15	PSME2, PSMD3, DDX17, RANBP2, PSMB4, KHDRBS1	11
16	DCTN3, EIF4A1, PLG, DMTF1, ITSN2, STAT6, YWHAZ, PSME1, NFKBIA, VWF, PAFAH1B1, TAF1, IRF9	10

**Table 3:** Total 42 hub proteins of pathogen i.e. *L. interrogans*.

Rank	Proteins	Degree
1	FusA	42
2	ClpB	30
3	PurC	24
4	GroL	22
5	GatA	21
7	RpoD	19
8	PyrG	18
9	CarB, GltX, GpmI	17
10	UvrB, GlyA, DnaJ, HisD	16
11	ArgS, Eno, MetK, Tuf	15
12	AsnS, UvrA, ArgG	14
13	CarA, DnaK, ProS	13
14	MetG, PurT, LeuS, DinB, ProA	12
15	IleS, LysS, LeuD, GcvP, PurD, GlmS	11
16	PyrB, ArgF, PrfB, PrfA, AlaS, SerS, GlyQS	10

### Sub-cellular localization of proteins

To identify sub-cellular localization of all bacterial proteins participating in the interactions with host proteins, all 145 bacterial proteins were subjected to the prediction of their sub-cellular localization as discussed in the methods section and the detailed results are available in Supplementary Table S1. After prediction, it was found that majority of bacterial proteins participating in PHPIs were cytoplasmic proteins (CPs, 128) followed by outer membrane proteins (OMPs, 9), inner membrane proteins (IMPs, 4) and periplasmic proteins (PPs, 4). The sub-cellular localizations of all proteins are summarized in the Table 4.

**Table 4:** Sub-cellular localization distribution of *L. interrogans* proteins.

Localization	Number of proteins, LIC
Outer membrane	9
Periplasmic	4
Inner membrane	4
Cytoplasmic	128

**Figure 6:**
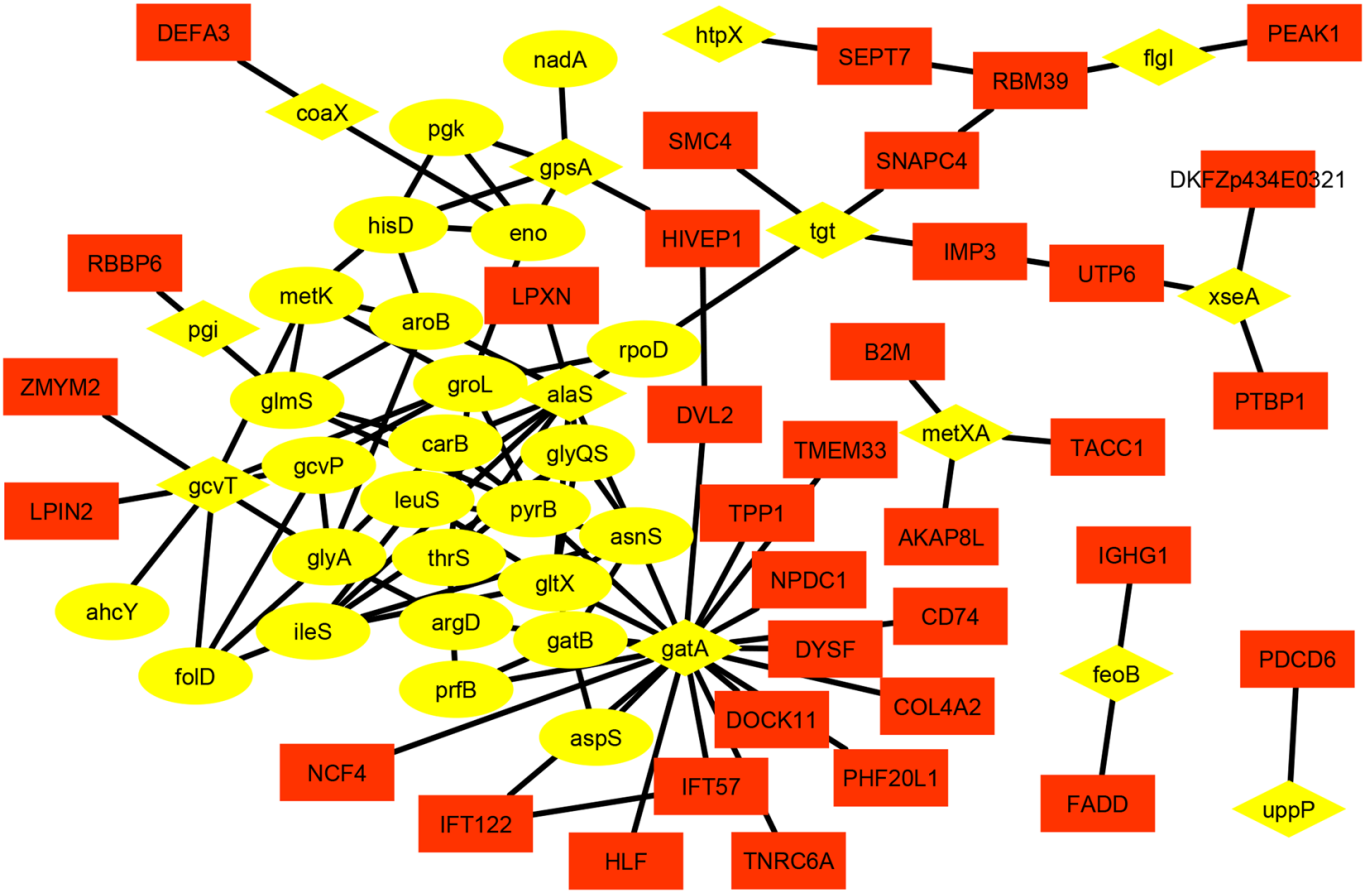
Sub-network of 13 membrane proteins and their first neighbors from the whole PHPIs network. Diamond shaped yellow colored nodes indicate 13 membrane proteins including 9 outer and 4 inner membrane proteins of *L. interrogans*, oval shaped yellow colored nodes indicate 25 other including cytoplasmic and periplasmic proteins of *L. interrogans*, and rectangular red colored nodes indicate 35 human proteins. Here, these oval and rectangular shaped nodes are the first neighbors of diamond shaped nodes.

Further, out of 638 proteins (nodes) with 586 interactions, a total of 48 proteins having 35 interactions among themselves, were retrieved based on their sub-cellular localization which could be considered as putative and credible PHPIs. Out of these 48 proteins participating in these interactions, total 13 bacterial membrane proteins (9 outer and 4 inner membrane) were found to be interacting with a total of 35 human proteins (as their first neighbors, Fig. 6) as visualized by Cytoscape^52^. The complete details of 35 interactions involving these 13 bacterial membrane proteins (MPs) and 35 human proteins with their biological processes have been listed in Table 5.

**Table 5:** Top 35 LIC-human protein-protein interactions.

LIC Gene	LIC Protein Official full name	Human gene name	Human Protein Official full name	Biological process
alaS	Alanine–tRNA ligase	LPXN	Leupaxin	cell adhesion, negative regulation of B cell receptor signaling pathway
coaX	Type III pantothenate kinase	DEFA3	Neutrophil defensin 3	antimicrobial humoral response
feoB	Fe(2+) transporter FeoB	FADD	FAS-associated death domain protein	activation of cysteine-type endopeptidase activity
		IGHG1	Immunoglobulin heavy constant gamma 1	B cell receptor signaling pathway
flgI	Flagellar P-ring protein	RBM39	RNA-binding protein 39	mRNA processing
		PEAK1	Pseudopodium-enriched atypical kinase 1	cell migration
gatA	Glutamyl-tRNA(Gln) amidotransferase subunit A	CD74	HLA class II histocompatibility antigen gamma chain	activation of MAPK activity, antigen processing and presentation of endogenous antigen
		DOCK11	Dedicator of cytokinesis protein 11	B cell homeostasis
		IFT122	Intraflagellar transport protein 122 homolog	camera-type eye morphogenesis, iliary receptor clustering involved in smoothened signaling pathway
		PHF20L1	PHD finger protein 20-like protein 1	regulation of transcription, DNA-templated
		DVL2	Segment polarity protein dishevelled homolog DVL-2	beta-catenin destruction complex disassembly
		TPP1	Tripeptidyl-peptidase 1	bone resorption, central nervous system development
		DYSF	Dysferlin	muscle contraction, plasma membrane repair
		COL4A2	Collagen alpha-2(IV) chain	Angiogenesis
		TMEM33	Transmembrane protein 33	cellular protein localization
		NCF4	Neutrophil cytosol factor 4	antigen processing and presentation of exogenous peptide antigen via MHC class I, TAP-dependent
		HLF	Hepatic leukemia factor	positive regulation of transcription from RNA polymerase II promoter
		TNRC6A	Trinucleotide repeat-containing gene 6A protein	regulation of gene silencing by miRNA
		NPDC1	Neural proliferation differentiation and control protein 1	regulation of immune response
		IFT57	Intraflagellar transport protein 57 homolog	activation of cysteine-type endopeptidase activity involved in apoptotic process
gcvT	Aminomethyltransferase	LPIN2	Phosphatidate phosphatase LPIN2	fatty acid catabolic process
		ZMYM2	Zinc finger MYM-type protein 2	cytoskeleton organization
gpsA	Glycerol-3-phosphate dehydrogenase	HIVEP1	Zinc finger protein 40	multicellular organism development, signal transduction
htpX	Protease HtpX homolog	SEPT7	Septin-7	cell differentiation
metXA	Homoserine O-acetyltransferase	B2M	Beta-2-microglobulin	antibacterial humoral response
		AKAP8L	A-kinase anchor protein 8-like	cell cycle G2/M phase transition, mitotic chromosome condensation
		TACC1	Transforming acidic coiled-coil-containing protein 1	cell division
pgi	Glucose-6-phosphate isomerase	RBBP6	E3 ubiquitin-protein ligase RBBP6	cellular response to DNA damage stimulus, DNA replication
tgt	QueuinetRNA-ribosyltransferase	SMC4	Structural maintenance of chromosomes protein 4	cell division, kinetochore organization
		SNAPC4	snRNA-activating protein complex subunit 4	cell differentiation
		IMP3	U3 small nucleolar ribonucleoprotein protein IMP3	rRNA processing
uppP	Undecaprenyl-diphosphatase	PDCD6	Programmed cell death protein 6	activation of cysteine-type endopeptidase activity involved in apoptotic process
xseA	Exodeoxyribonuclease 7 large subunit	DKFZp434E0321	Putative uncharacterized protein DKFZp434E0321	cell adhesion
		UTP6	U3 small nucleolar RNA-associated protein 6 homolog	maturation of SSU-rRNA from tricistronicrRNA transcript (SSU-rRNA, 5.8S rRNA, LSU-rRNA), rRNA processing
		PTBP1	Polypyrimidine tract-binding protein 1	fibroblast growth factor receptor signaling pathway

LIC proteins are referenced according to their UniProt/SwissProt protein coding gene and protein name (columns 1, and 2 respectively). Human proteins are referenced with their cognate gene name and their protein official full name with their main biological functions (columns 3, 4 and 5 respectively).

### Conservation of MPs and PPs among *Leptospira* spp

In pathogenic bacteria, MPs or especially OMPs are the most promising therapeutic or vaccine candidates, as these are likely to interact with the host immune cells^53^. Hence, the identification of conserved MPs among all spp. of *Leptospira* is crucial for reliable and rapid identification of potential vaccine candidates. For this, the proteome sequences from 21 *Leptospira* spp. (including 9 pathogenic, 6 intermediate and 6 saprophytic spp.) were screened for homologs of the 9 outer membrane, 4 inner membrane, and 4 periplasmic proteins as described in the methods section. Interestingly, all the pathogenic spp. of *Leptospira* contained homologs of these 9 outer membrane, 4 inner membrane and 4 periplasmic proteins (Supplementary Table S2). Of the 9 OMPs, only GpsA (P61742) and MetXA (Q72R95) were present in all 21 *Leptospira* spp. (pathogenic, intermediate and saprophytic) while, inner membrane protein CoaX (Q72NP0) and outer membrane protein Tgt (Q72TL3) were present in only pathogenic and intermediate but absent in saprophytic *Leptospira* spp. Two outer membrane proteins *viz*. GcvT (Q72VI6) and GatA (Q72SC3) were present in pathogenic and intermediate except *L. wolffii*; while, outer membrane protein FlgI (Q72SP8) was present in all pathogenic and intermediate except *L. licerasiae*, *L. wolffii* and *L. venezuelensis*. However, total three inner membrane proteins *viz*. FeoB (Q72SI0), Htpx (Q75FP1), UppP (P62465), and three outer membrane proteins *viz*. XseA (Q72RZ7), Pgi (Q72MT7) and AlaS (P61703) were present in only pathogenic but absent in both intermediate and saprophytic. Moreover, out of 4 periplasmic proteins, only Flab (Q72R59) and GlyA (Q72PY2) were present in all pathogenic, intermediate and saprophytic *Leptospira* spp.; while, GpmI (Q72VB8) was absent in saprophytic and PurD (Q72V31) in both intermediate and saprophytic.

### Functional enrichment analysis

Functional annotations of proteins are required to understand their molecular functions and biological processes. Several previous studies have indicated that surface and membrane proteins play a key role in course of interaction of pathogen with its host^54–56^. All proteins associated with the human immune system also contribute significantly to the pathogen-host interactions^57,58^.

In order to investigate the molecular functions and biological processes associated with these proteins, functional enrichment analysis was performed. After performing the functional enrichment analysis of all human proteins present in the PHPIs network, all 493 proteins of human were classified into 11 clusters. The significantly enriched gene ontology (GO) terms (*p*-value < 0.05) in these proteins interacting with leptospiral proteins were chosen to be important to understand the infection mechanism. These GO terms point out the biological processes, molecular functions and pathways associated with pathogen targeted human proteins.

#### Biological process

In total 117 significantly enriched GO terms (*p*-value <0.05) for biological process (details available in Supplementary Table S3) were found to be associated with 493 host proteins involved in pathogen-host interaction. The significantly enriched process related GO terms of human proteins interacting with bacterial proteins are important to unravel the infection mechanisms. The first 20 enriched terms are listed in Table 6 to highlight the human processes that are attacked by pathogens during infection. By analyzing the data, it was noticed that majority of proteins were involved in processes like apoptotic process (31), immune response (25), cell-cell adhesion (23), intra-cellular signal transduction (19), cell proliferation (17), processing and presentation of antigen (13), and T cell receptor signaling pathway (12).

**Table 6:** First 20 enriched GO process terms in human proteins targeted by bacteria.

GO ID	GO Process term	Count	*p*-value
GO:0002474	antigen processing and presentation of peptide antigen via MHC class I	11	2.12E-09
GO:0002480 GO:0002479	antigen processing and presentation of exogenous peptide antigen via MHC class I, TAP-independent	7 13	2.17E-08 5.73E-08
GO:0060333	interferon-gamma-mediated signaling pathway	12	1.80E-06
GO:0098609	cell-cell adhesion	23	2.07E-06
GO:0006397	mRNA processing	17	1.56E-05
GO:0019882	antigen processing and presentation	9	7.47E-05
GO:0006915	apoptotic process	31	1.55E-04
GO:0060337	type I interferon signaling pathway	9	2.22E-04
GO:0006457	protein folding	15	2.33E-04
GO:0001916	positive regulation of T cell mediated cytotoxicity	5	2.55E-04
GO:0016032	viral process	20	2.78E-04
GO:0043066	negative regulation of apoptotic process	26	3.08E-04
GO:0045087	innate immune response	25	3.26E-04
GO:0071260	cellular response to mechanical stimulus	9	4.56E-04
GO:0000122	negative regulation of transcription from RNA polymerase II promoter	35	4.78E-04
GO:0030036	actin cytoskeleton organization	12	5.27E-04
GO:0032355	response to estradiol	10	5.43E-04
GO:0050690	regulation of defense response to virus by virus	6	6.57E-04
GO:0034976	response to endoplasmic reticulum stress	9	6.62E-04
GO:0017148	negative regulation of translation	8	6.79E-04

#### Molecular function

In the present study, total 46 significantly enriched molecular function related GO terms (*p*-value < 0.05) were found to be associated with 493 human proteins involved in pathogen-host interaction (details available in Supplementary Table S4). The first 20 enriched terms are listed in Table 7 to reveal the molecular functions of human proteins that used to get altered by pathogens during infection. The result showed that maximum numbers of proteins (336) were involved in protein binding, followed by 77 in poly (A) RNA binding and 34 in homo-dimerization activity of protein. Whereas, 24 proteins were associated with cadherin binding involved in cell-cell adhesion and protein kinase binding, 19 with transcription factor binding, 18 with ubiquitin protein ligase binding and other enzyme binding and 10 with antigen binding.

**Table 7:** First 20 enriched GO function terms in human proteins targeted by bacteria.

GO ID	GO function term	Count	*p*-value
GO:0005515	protein binding	336	2.09E-24
GO:0044822	poly(A) RNA binding	77	2.84E-14
GO:0042802	identical protein binding	46	2.42E-07
GO:0098641	cadherin binding involved in cell-cell adhesion	24	2.95E-06
GO:0042605	peptide antigen binding	8	6.27E-06
GO:0001948	glycoprotein binding	11	7.31E-06
GO:0043621	protein self-association	8	1.88E-04
GO:0019901	protein kinase binding	23	4.45E-04
GO:0008134	transcription factor binding	19	5.78E-04
GO:0050681	androgen receptor binding	7	5.89E-04
GO:0003823	antigen binding	10	0.001620667
GO:0044212	transcription regulatory region DNA binding	15	0.001630945
GO:0031625	ubiquitin protein ligase binding	18	0.001695178
GO:0005201	extracellular matrix structural constituent	8	0.001901561
GO:0042803	protein homodimerization activity	34	0.001952651
GO:0046977	TAP binding	3	0.002052872
GO:0003756	protein disulfide isomerase activity	5	0.002409702
GO:0003713	transcription coactivator activity	16	0.002546855
GO:0051082	unfolded protein binding	10	0.002551929
GO:0030881	beta-2-microglobulin binding	4	0.002580398

#### KEGG pathway

A total of 37 significantly enriched KEGG pathway terms (*p*-value < 0.05) were found to be associated with these 493 human proteins (details available in Supplementary Table S5). The first 20 enriched terms are listed in Table 8 to highlight the significant pathways associated with these human proteins which get manipulated and hampered by the pathogens during infection. After analyzing the enrichment data, it was inferred that majority of the host signaling pathways which are disrupted during the disease pathogenesis, are pathways related to Antigen processing and presentation (15), Endocytosis (15), Phagosome (14), Focal adhesion (12), T cell receptor signaling pathway (8), NOD-like receptor signaling pathway (7), and B cell receptor signaling pathway (7).

**Table 8:** First 20 enriched KEGG pathway terms in human proteins targeted by bacteria.

Pathway ID	Term	Gene count	*p*-value
hsa04612	Antigen processing and presentation	15	8.31E-09
hsa05416	Viral myocarditis	12	2.13E-07
hsa05169	Epstein-Barr virus infection	19	2.37E-06
hsa05168	Herpes simplex infection	18	6.02E-06
hsa05203	Viral carcinogenesis	19	6.99E-06
hsa05134	Legionellosis	9	7.62E-05
hsa05140	Leishmaniasis	10	9.52E-05
hsa05166	HTLV-I infection	19	1.37E-04
hsa04145	Phagosome	14	1.93E-04
hsa05332	Graft-versus-host disease	7	1.94E-04
hsa05330	Allograft rejection	7	3.73E-04
hsa04520	Adherens junction	9	5.31E-04
hsa04940	Type I diabetes mellitus	7	7.54E-04
hsa05161	Hepatitis B	12	0.001542118
hsa05212	Pancreatic cancer	8	0.001550511
hsa05320	Autoimmune thyroid disease	7	0.00235216
hsa04622	RIG-I-like receptor signaling pathway	8	0.002389704
hsa04621	NOD-like receptor signaling pathway	7	0.003137327
hsa04514	Cell adhesion molecules (CAMs)	11	0.004296064
hsa05210	Colorectal cancer	7	0.005713384

## Discussion

The pathogen-host protein interactions (PHPIs) play an important role during the invasion of host immune systems by bacteria for its persistence and replication within host^37,59^. Nevertheless, the understanding of leptospiral pathogenesis and virulence is limited in spite of several *in vitro* studies made therein to understand pathophysiology of *L. interrogans*, including leptospiral protein binding to different components of plasma^13,60^, extracellular matrix, and vascular endothelial cadherin of host, which contributes to the dissemination of bacteria leading to hemorrhagic manifestations^61^. Hence, in the scarcity of experimentally-verified pathogen-host interactions (PHIs) data, identification of pathogen-host protein interactions (PHPIs) using computational methods is worthwhile to enlighten the infection mechanisms.

A systems level understanding of interactions between pathogen and host proteins is a crucial step to establish a relationship between pathogen and host. In this regard, for understanding the pathogen-caused changes at proteome level and thereby alteration in related metabolic pathways during the course of infection, protein interactions mapping between pathogen and host is a most important factor^62^. Biological networks i.e. protein or gene networks can be employed to reveal underlying mechanism and properties of complex disease systems^63^. Advancements in computational algorithms for the analysis of protein interactions data may further facilitate in unraveling the underlying mechanism of bacterial pathogenesis. Thus, the present study is an attempt towards the exploration of infection strategies used by *Leptospira* based on the systematic analysis of pathogen-host protein interactions network. The methodology implemented in our study was also previously employed to decipher PHPIs of Mycobacterium tuberculosis^31,32^, Hepatitis C Virus^64^ and Human Papilloma Virus 16^65^ with human. The presented network biology approach of reconstructing the pathogen-human interactions network in terms of topological properties of the network and functional annotations of proteins involved in pathogen-host interactions is in good agreement with the observations previously reported. However, the framework applied to explore the molecular basis of pathogenesis needs proper validation to bottom up high confidence and reliability on the predicted set of PHPIs for the development of immunotherapeutic targets.

The development, mapping and analysis of protein-protein interactions (PPIs) either intra-species or inter-species is critically important to understand the complex biological processes^66–68^. It helps us to identify novel or putative proteins and their interactions suitable for the intervention of molecular therapeutics. A subtle perturbation in a biological i.e. gene or protein interactions network can produce disease phenotypes^44^. Herein, the thorough interactome mapping strategy with proteome scale coverage at species level allowed us to explore the differences and commonalities between inter- and intra-species protein networks for unraveling the infection mechanism. Thus, the subsequent analyses provide key insights about their cellular process disruption within host cells during infection.

The inter-species PPIs between *L. interrogans* and human were predicted and inferred by employing the approach of “interolog”. Consequently, the intra-species PPIs among *L. interrogans* and human were also retrieved to complement the PHPIs. By integrating all data, a PHPIs network comprising 1538 interactions among 145  leptospiral proteins and 510 human proteins was constructed. Out of these 1538 interactions of the PHPIs network, there is a total 586 pathogen-host protein interactions between 145 leptospiral proteins and 493 human proteins. As these interactions data have been produced with computational prediction methods, which are prone to false positive, it was needed to assess the quality of these data. For assessing the predicted data quality, we calculated the value of specificity by using true negative data as predicted by our method used for the predictions of PHPIs. The specificity of the data was found to have a very high value which indicates that our predicted results have very high reliability in terms of accuracy. We also compared our predicted data with previously reported data for assessing sensitivity and accuracy of the data. For which we found 25 *L. interrogans* serogroup Icterohaemorrhagiae serovar Copenhageni (strain Fiocruz L1-130) (LIC) proteins having significant role in the survival and infection as reported in previous study of Mehrotra *et al*.^43^. As the coverage and accuracy of our predicted interactions were dependent on the previously reported experimental data on pathogen-host protein interactions. The accuracy and coverage would be increased with the increasing number of these identified template interactions. Recently, protein structures based PPIs were predicted between *L. interrogans* and human^43^. The predictions based on protein structures possibly could exclude true negatives, though it also had limitation in terms of the number of known complexes of proteins. Furthermore, a method based on time-course microarray data was developed for the experimental identification of PHPIs instead of only based on the previously known templates^69^. While this method would make biological sense, it may not be convenient and easy for all the species. Overall, each method in some aspects would have a good performance but some limitations too.

The methodology employed here to predict the PHPIs and subsequent analysis have some limitations in terms of well characterized proteome data of *L. interrogans*. As discussed in the methods section, majority of leptospiral proteins are un-reviewed and not well-characterized. For example, major surface proteins of *Leptospira* such as LipL32, LipL46 and LigB which are un-reviewed proteins and hence, the interactions predicted here do not have involvement of these proteins. While, LigA protein, which is a well characterized and annotated, is showing interactions with key human proteins in our results (as discussed later in this section). Thus, the coverage of our predicted results could have been increased with the availability of more annotated and well characterized leptospiral proteins.

To check the validity of these predicted PHPIs, it was also needed to investigate whether the distributions of degree, betweenness centrality (BC), clustering coefficient (CC) and eigenvalues of the PHPIs network would be similar to that of the intra-species networks. Hence, the above-mentioned analyses were performed with protein sets involved in inter-species interactions as well as intra-species interactions of all the interacting proteins. The degree distribution, clustering coefficient, betweenness centrality and eigenvalue distributions of the inter-species i.e. PHPIs network showed commonality in comparison with the intra-species networks except the number of cliques, which was higher in case of intra-species networks compared to the inter-species (PHPIs) network and reflects its perturbed nature (Table 1). The degree distributions of all networks follow the power law and have scale free nature, which indicated the presence of hub nodes in these networks. It has been noticed that bacterial proteins favorably interact with hub and bottleneck proteins of the host protein network^37,59^. However, attacking high degree nodes (hubs) even in small numbers can alter the network functionality significantly by altering the organization and subsequently topology of the network^70,71^. When the bacterial proteins interact with the host proteins, the organization of host protein network gets disrupted by depletion in the number of cliques thus by resulting to the perturbed system. This perturbed nature of the PHPIs network could be the leading cause of bacterial pathogenesis and the development of disease condition.

*L. interrogans* is an extracellular pathogen. Albeit, recent studies have demonstrated the intracellular fate of pathogenic *Leptospira* in human and mouse macrophages^72,73^. In our predicted PHPIs, out of 145 participating proteins of LIC, a total of 128 of LIC proteins have been predicted to be localized in the bacterial cytoplasm. Out of 17 remaining proteins, 9 proteins were predicted as outer membranes, 4 as periplasmic and 4 as inner membranes. Recent experimental studies have revealed that the endogenous or cytoplasmic proteins are moonlight proteins because of their crucial roles in disease pathogenesis such as survival, evasion, transmission etc. besides their other roles. When leptospiral components including cytoplasmic proteins get exposed due to bacterial lysis caused by host immune response, may interact with host proteins and have role in pathophysiology of the disease via triggering inflammatory response such as increased production of TNF and IL-6^74^, secretion of leukotriene B_4_, prostaglandin E_2_, and nitric oxide^75^ or causing direct injury^76^. In the context of this, it has been shown that the alteration or inhibition of Na/K-ATPase caused by leptospiral GLP can trigger the inflammatory cascade^77,78^ thereby leading to the exacerbation of multi-organ dysfunction associated immune response and resulting to acute renal, lung^79,80^ and liver failure^77,81^. In pathogenic spp. of *Leptospira*, the proteins encoded by *lig* genes have been found present during infection within mammals^82^. Several previous studies have reported the binding of two proteins of this family, LigA and LigB, with host molecules like Factor H (FH)^83^, C4b-binding protein (C4BP)^83^, and Plasminogen (PLG)^84^. Leptospiral interaction with human’s fibrinolytic system by capturing surface plasminogen (PLG) and subsequent plasmin (PLA) generation facilitates host endothelial cell penetration and invasion^85,86^. Our study also showed the interaction of LigA with total seven human proteins *viz*. BAG6, ELF1, MLLT6, TP53BP2, PITPNM3, ITSN2 and MID2. Out of these seven interactor proteins, one (BAG6) is associated with protein binding and ubiquitin protein ligase binding, three (ELF1, MLLT6 and ITSN2) with protein binding, one (TP53BP2) with NF-kappaB binding and P53 binding, one (PITPNM3) with protein and lipid binding and one (MID2) with protein homodimerization activity and microtubule binding as enriched by molecular function GO terms. Further, the protein LepA-σ54 has been found to be playing an important role in the survival of *L. interrogans* within the host as reported by Fouts *et al*.^87^. As per our predicted PHPIs, protein encoded by leptospiral gene *LepA* interacts with total eight human proteins *viz*. TRPC1, SRSF3, HSPE1, TBX6, ARRB1, CDR1, ANKRD12 and BTBD2. Out of these eight human proteins, one (TRPC1) is associated with protein binding, one (SRSF3) with protein and nucleotide binding, one (HSPE1) with chaperone binding, two (TBX6 and CDR1) with protein binding, one (ARRB1) with protein binding, transcription factor binding, transcription regulatory region DNA binding, ubiquitin protein ligase binding, estrogen receptor binding, histone acetyltransferase activity and AP-2 adaptor complex binding, and one (BTBD2) with protein binding and ubiquitin protein ligase binding as enriched by molecular function GO terms. The protein encoded by the gene *ClpB* is not only responsible for the survival of *L. interrogans* under stressed conditions such as the oxidative and thermal, but also for causing and developing infection within the host^88,89^. The predictions made in our study have revealed that the protein ClpB is interacting with total 24 human proteins *viz*. STAM2, TXNIP, EEF1A1, BNIP3L, TRPS1, H1FX, PLG, COL4A1, HLA-A, TRPC1, CAP1, AES, MVP, NCOA1, PIK3AP1, MGA, JAKMIP1, CCDC115, CHRD, POT1, SLTM, BIN2, DKFZp434E0321 and DMTF1. Out of these interactors of ClpB, COL4A1 is associated with extracellular matrix structural constituent as enriched by molecular function and biological process GO terms. While, thirteen proteins (STAM2, PLG, EEF1A1, TRPS1, TRPC1, MVP, MGA, CHRD, POT1, BIN2, PIK3AP1, BNIP3L and CCDC115) are associated with protein binding, one (CAP1) with actin cytoskeleton organization, one (TXNIP) with ubiquitin protein ligase binding, one (AES) with transcription corepressor activity, one (NCOA1) with androgen receptor binding, and four (H1FX, HLA-A, JAKMIP1 and SLTM) with RNA binding as enriched by molecular function GO terms. A recent study by Dong *et al*. showed that the leptospiral proteins encoded by genes *HslU* and *HslV* form a complex comprising ATP-dependent chymotrypsin-like threonine peptidase-type AAA+ chaperone and Ntn peptidase. This complex has a contribution not only in the survival and virulence of the pathogen during host infection but also in the transmission of the disease^90^. The protein interactions inferred in our study showed that the leptospiral protein HslU interacts with only one human protein *i.e*. ING5. As per our functional annotation, it was noticed that ING5 is associated with biological process terms like negative regulation of cell proliferation, regulation of signal transduction by p53 class mediator and positive regulation of apoptotic process and molecular function term like protein binding.

All bacterial membrane proteins (outer as well as inner membrane) interacting with host proteins were found to have their orthologs in all pathogenic spp. of *Leptospira* including two outer membrane proteins (GpsA and MetXA) with their orthologs in all spp. of *Leptospira* including pathogenic, intermediate and saprophytic (Supplementary Table S2). In Gram-negative bacteria, outer membrane proteins (OMPs) have been found to perform diverse functions including its involvement in the pathogen-host interactions^91^. Membrane proteins (MPs) facilitates the adherence of *Leptospira* to endothelial cell surfaces of host via binding to VE-cadherin, and lead to vascular endothelial damage, consequently facilitating the pathogen escape to different tissues and hence contribute to the hemorrhagic manifestations of the disease^61^. In present PHPIs network, it was noticed that the protein encoded by gene *gpsA* is targeting HIVEP1, a zinc finger protein which is involved in signal transduction. Whereas, the protein encoded by *metXA* is targeting three host proteins viz. B2M (Beta-2-microglobulin), AKAP8L (A-kinase anchor protein 8-like) and TACC1 (Transforming acidic coiled-coil-containing protein 1). Among these three target host proteins, the protein B2M is involved in antibacterial humoral response; AKAP8L in cell cycle G2/M phase transition and mitotic chromosome condensation; and TACC1 in cell division. Thus, by detailed analysis of target proteins of these bacterial MPs, it can be inferred that how these MPs are involved in the manipulation of essential cell mechanism of hosts and thereby disruption of the metabolic pathways.

The major strategy of bacterial infection is through evasion or suppression of host immune responses by attacking human proteins^59,92–94^. Likewise, from the functional enrichment analysis of bacteria targeted human proteins in our work, it could be concluded that bacteria use to attack host proteins of metabolic pathways and immune systems. Also, our analysis showed involvement of the bacterial proteins (such as ClpB, TrpB, SecA, LeuB, CarA, FusA and PurC) in inactivation of T cells, adaptive and innate immunity and inflammation thereby resulting to damage of the defense mechanism of the host. Previously, it was reported that bacterial proteins interact with LCK and NF-κB^59,95,96^ to disrupt the mechanisms of T cell responses and inflammation; to interact with toll-like receptors (TLR2, TLR4 and TLR7)^97–99^ to crumple the host immune system, which are the key players of adaptive and innate immunity. In our data, several other proteins are also involved in interaction with pathogen and have a role in immunity in humans. The systematic exploration of bacterial and host proteins involved in pathogen-host interactions via metabolic processes and molecular functions can help to draw a complete picture of bacterial pathogenesis and will help to identify drug target or vaccine candidate.

Bacteria have a tendency to interact with human proteins enriched in the regulation of metabolic processes in addition to cellular processes. In our study, enzyme involved in lipid metabolism *e.g*. Carnitine palmitoyltransferase IA (CPT1A) was also identified as targets of bacterial proteins (CarA and LeuD). The inhibition of CPT1A expression results in lipid accumulation in liver due to alteration in oxidation of plasma fatty acid, thereby leading to organ dysfunction^100,101^. The bacterial proteins targeting these human proteins may be the reason behind the alteration of molecular functions and biological processes resulting to the disrupted cellular mechanism of host.

## Conclusion

A total of 586 pathogen-host protein interactions between 145 proteins of *Leptospira* and 493 proteins of human were identified and analyzed using a network theory approach combined with *in silico* algorithms and functional annotations. The analyses and annotations of these interactions facilitated the effective understanding of pathogenesis and host immune response leading to the disease development. Of these, 35 interactions between 13 leptospiral membrane proteins and 35 human proteins were termed as ‘putative and credible’ interactions based on bacterial protein sub-cellular localization. These outer and inner membrane proteins were found to have their orthologs in all pathogenic species; while, some of them were also found to have their orthologs among intermediate and saprophytic species. Further, our systematic protein network and their functional enrichment analyses concluded that bacteria interact with the human proteins majorly involved in the immune systems and metabolic processes as its main infection strategy via the involvement of bacterial membrane proteins. These bacterial membrane proteins play a vital role in the manipulation of cellular processes within host and thereby causing infectious diseases. Thus, these findings signify that the proteins participating in such interactions hold immense potential to serve as effective immunotherapeutic candidates for vaccine development and provide apt avenues for the disease intervention.

## Materials and Methods

To understand the development and progression of leptospirosis, we predicted the pathogen-host protein interactions and subsequently inferred the infection mechanism of *L. interrogans*.

### *L. interrogans* proteome data

Icterohemorrhagic serogroup of *L. interrogans* is the widely studied serogroup and it is reported in most of the cases of the disease, hence the strain Fiocruz L1-130 of *L. interrogans* serogroup Icterohaemorrhagiae serovar Copenhageni (LIC) was considered for the pathogen-host protein interactions (PHPIs) study. To predict the PHPIs, all proteins of this strain were extracted from the UniProtKB^102^. In UniProtKB, majority of proteins of *Leptospira* is hypothetical and without any characterized function. Hence, to keep the authenticity of the data and to avoid any bias to the analysis, only literature authenticated and reviewed proteins were taken into account for analysis purpose. Thus, a total of 374 reviewed proteins of LIC were considered for the predictions of PHPIs.

### Inter-species interactome

For predicting the pathogen-host protein interactions, BLASTP (https://blast.ncbi.nlm.nih.gov/Blast.cgi?PAGE=Proteins) was performed with the help of Host Pathogen Interaction Database (HPIDB) v2.0^103^ and Biana Interolog Prediction Server (BIPS)^104^ against all known pathogen-host protein interactions using cut-off 1e-10 E-value, 30% minimum identity and 90% query coverage. HPIDB and BIPS implement the method of Interolog to predict pathogen-host protein interactions. Interologs are the proteins which preserve their property and ability of interactions during course of evolution. If A and B are two proteins such that A interact with B, while A’ and B’ are homologs of A and B respectively then A’ must interact with B’ (Fig S1).

The predicted inter-species interactions data for *Leptospira-*human (LIC-HSA) contained 1201 nodes with 1198 edges. Further, all the UniProtKB AC/IDs corresponding to pathogen as well as host were mapped to their respective gene symbols. The mapping was performed in order to unify protein AC/IDs and to eliminate the redundancy of interactions due to the existence of different isoforms of a single protein corresponding to one gene. Thus, the gene symbol was used to identify the proteins during analysis and interpretation. The mapped PHPIs data for LIC-HSA contained a total of 638 nodes with 1137 edges. Duplicate edges and self-loops were removed from the data and hence, the final processed interactions (i.e. only non-redundant) contained total 638 nodes with 586 edges.

### Domain-domain interactions (DDIs)

Protein domains are important functional parts of a protein and help to facilitate protein-protein interactions between two proteins. Two proteins A and B must interact if domain ‘a’ of protein A interacts with the domain ‘b’ of protein B. Besides predicting PHPIs using HPIDB, protein interactions with having domain-domain interactions (DDIs) were derived from iPfam (http://ipfam.org) and 3DID (https://3did.irbbarcelona.org/) database integrated in BIPS.

### Intra-species interactome

To find intra-species interactions, all the proteins of human and *L. interrogans* found to be involved in inter-species interactions, were mapped to the STRING v10.5^105^. All experimentally determined and database curated protein interactions with minimum interaction score 0.4 were considered for the study. The extracted intra-species interaction networks of human and LIC were contained total 289 nodes with 705 edges for human and 115 nodes with 247 edges for LIC.

### Pathogen-host interaction network construction

There are two sets of interactions data *viz*. inter-species interactions (LIC-HSA) and Intra-species interactions (LIC and HSA). To get a holistic set of PHPIs, these both sets were merged using “union” operation of “Set Theory”. There were total 638 nodes with 586 edges for LIC-HSA, total 115 nodes with 247 edges for LIC and total 289 nodes with 705 edges for HSA; and after merging, the merged network contained total 655 nodes with 1538 edges.

### Network randomization, validation and quality assessment

To check whether the interactions between the proteins of pathogen (*L. interrogans*) and host (human) and the resultant PHPIs network is statistically and biologically valid or not, randomized networks were constructed as a negative control for the hypothesis. A network randomization approach called Erdos-Renyi (ER)^106^ was employed by preserving the set of all nodes and the interactions present in the real network. For this, “igraph” package^107^ implemented in R statistical computing environment (https://www.r-project.org/) was used to construct random networks for 100000 times and the average degree distribution was calculated to compare with the degree distribution of the real network.

These predicted PHPIs data were also subjected for their quality assessment. For doing so, first a data set of non-interacting pairs of proteins were retrieved from the Negatome v.2.0 database (http://mips.helmholtz-muenchen.de/proj/ppi/negatome). The Negatome is a database of unlikely engaged proteins and domains of proteins in physical interactions. The data contained within this database is derived by curating literature manually and also by analyzing all available three-dimensional (3D) structures of protein complexes^42^. These non-interacting pairs of proteins were considered as reference data set for the predictions of true negatives by employing the same approach which was used for the predictions of PHPIs. Second, the value of specificity was calculated. Further, the sensitivity and accuracy of our predicted data were also assessed by comparing them with previously reported PHPIs between *L. interrogans* and human.

### Structural properties of networks

Several topological measures have been proposed to explore the specific features of a network^108^ for assessing its validity as computationally predicted data are prone to false positives. Topological parameters like degree, degree distributions, clustering coefficients, betweenness and eigenvalue distributions were calculated for all the networks using the “igraph” R package.

#### Degree

It is the most basic structural property of a network. The degree of a particular node can be defined as the total number of neighbors of the particular node has.

#### Degree distribution

It denotes the fraction of vertices having degree k in a network.

#### Clustering coefficient

It is defined as ratio of the total number of connections a node is having by the possible number of connections that node can have.

#### Betweenness

The betweenness centrality of a node is the total number of shortest paths between node pairs passing through the node of interest divided by the total number of shortest paths between that node pairs.

#### Eigenvalue

It is defined as the number such that the determinant of a matrix minus the identity matrix multiplied by that number will be zero. In other words, it is defined as a set of values a parameter having for which, under a given condition, a differential equation will have a non-zero solution.

### Top connecting and hub nodes

Hub-nodes play important roles in structural and functional properties of a network. Hence, hub nodes present in the PHPIs network were detected based on the values of degrees of all nodes present in the network. These hub-nodes were termed as ‘top connecting nodes’ (TCNs). As hub-nodes play important role in the structural organization of a network, in case of protein networks these nodes may tend to form protein complexes or module like structures having important functional roles. Nodes having 10 or more than 10 connections were considered as hub-nodes.

### Predicting sub-cellular localization of proteins

All proteins of *L. interrogans* participating in pathogen-host interactions were mapped to their sub-cellular localization using the support vector machine implemented in the CELLO v.2.5 predictor^109,110^ based on n-peptide compositions. After depicting sub-cellular localization of bacterial proteins, the PHPIs were filtered based on the term “membrane” for further analysis and to increase the authenticity of the interaction data.

### Identification of protein homologs among *Leptospira* spp

These filtered interacting proteins were subjected to identify their homologs in the leptospiral proteome using protein BLAST based on the reciprocal best hit method as previously implemented by Grassmann *et al*.^9^. The best reciprocal hits of all protein sequences having similarity and coverage >70% and 90% respectively were considered as homologs (orthologs).

### Functional enrichment analysis

Functional enrichment analysis was performed for all host proteins participating in interactions by using the Database for Annotation, Visualization and Integrated Discovery (DAVID) v6.8^111^. Only terms enriched with *p*-value <= 0.05 were considered for the significance of the results. To identify the significant terms associated with each host protein, all three gene ontology (GO) terms *viz*. biological process, molecular function and KEGG pathway were scanned.

## Supplementary information

Supplementary Dataset 1

Supplementary Information
