# Author Correction: Inferring pathogen-host interactions between *Leptospira interrogans* and *Homo sapiens* using network theory

**DOI:** 10.1038/s41598-019-55326-0

**Published:** 2019-12-20

**Authors:** Swapnil Kumar, Kumari Snehkant Lata, Priyanka Sharma, Shivarudrappa B. Bhairappanavar, Subhash Soni, Jayashankar Das

**Affiliations:** Gujarat Biotechnology Research Centre, Department of Science & Technology, Government of Gujarat, Gandhinagar, 382011 India

Correction to: *Scientific Reports* 10.1038/s41598-018-38329-1, published online 05 February 2019

In Figure 5, proteins *viz*. HLA-A*03, HLA-B*1513, HLA-A*0226, HLA-A*33, HLA-A*02, and HLA-B*3512 of *H. sapiens* were inadvertently marked as hub proteins (red coloured nodes). The correct Figure 5 appears below as Figure 1.

**Figure 1:**
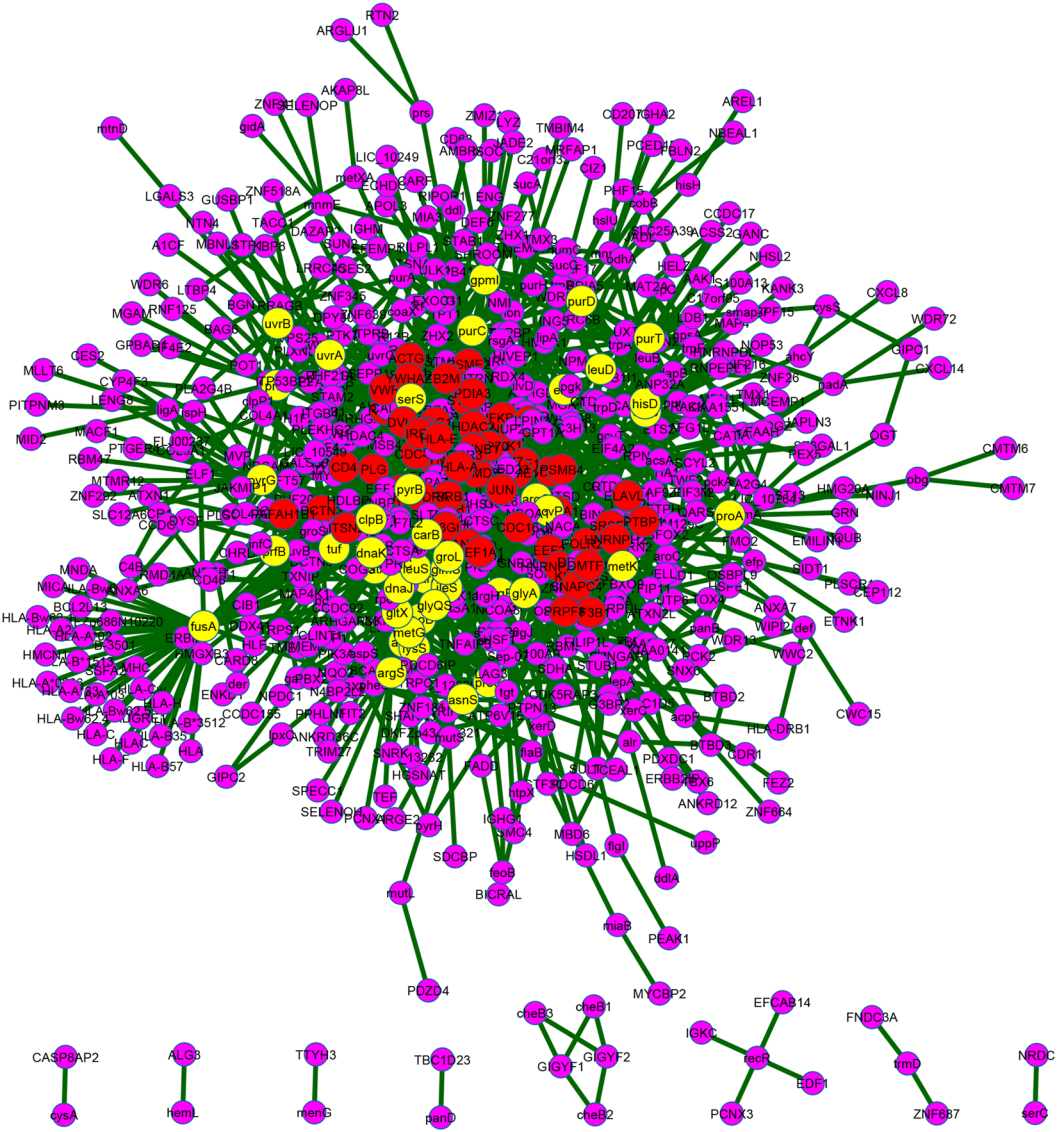
.

