# Supplementary material for: Inferring pathogen-host interactions between Leptospira interrogans and Homo sapiens using network theory
Source: Sci Rep. 2019 Feb 5;9:1434. doi: 10.1038/s41598-018-38329-1 (PMC6363727; doi:10.1038/s41598-018-38329-1)
Supplement: Supplementary file 2 — Supplementary Information [file 41598_2018_38329_MOESM2_ESM.docx]

**Inferring pathogen-host interactions between *Leptospira interrogans* and *Homo sapiens* using network theory**

**Swapnil Kumar, Kumari Snehkant Lata, Priyanka Sharma, Shivarudrappa B. Bhairappanavar, Subhash Soni, Jayashankar Das***

Gujarat Biotechnology Research Centre, Department of Science & Technology, Government of Gujarat, Gandhinagar – 382011, India.

**^*^**Corresponding author. Tel: +91 (079) 23252166; E-mail: [jayshankardas@gmail.com](mailto:jayshankardas@gmail.com)

**Figure S1. Homologous interactions inferred from interactions between homologs.** Protein A’ and B’ are interologs of protein A and B. Protein A and B have direct interactions, while protein A’ and B’ are their respective homologs. Hence, A’ will interact with B’ and the interaction between them is called homologous interaction.


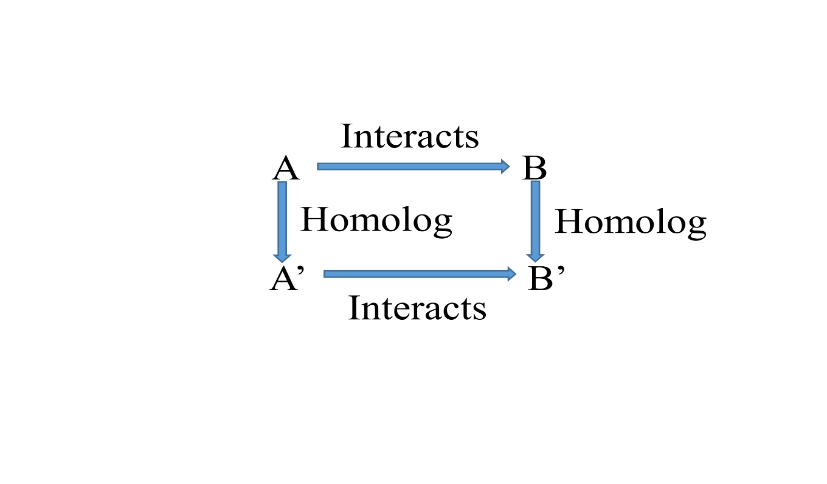


**Table S2. Conservancy of the 13 membrane proteins (MPs) and 4 periplasmic proteins (PPs) among 21 *Leptospira spp*.**

|  | **Protein ID^*^** | **Gene** | **Pathogenic *Leptospira* spp.** | **Intermediate *Leptospira* spp.** | **Saprophytic *Leptospira* spp.** |
| --- | --- | --- | --- | --- | --- |
|  |  |  | *ale. als. bor. kir. kme. may. nog. san. wei.* | *bro. fai. ina. lice. wolf. ven.* | *bif. mey. ter. van. wolb. yan* |
| Membrane Proteins | Q72NP0  Q72SI0  Q75FP1  P62465  P61742  Q72RZ7  Q72SP8  Q72MT7  Q72VI6  Q72SC3  Q72R95  P61703  Q72TL3 | coaX  feoB  htpX  uppP  gpsA  xseA  flgI  pgi  gcvT  gatA  metXA  alaS  tgt | ✓ ✓ ✓ ✓ ✓ ✓ ✓ ✓ ✓  ✓ ✓ ✓ ✓ ✓ ✓ ✓ ✓ ✓  ✓ ✓ ✓ ✓ ✓ ✓ ✓ ✓ ✓  ✓ ✓ ✓ ✓ ✓ ✓ ✓ ✓ ✓  ✓ ✓ ✓ ✓ ✓ ✓ ✓ ✓ ✓  ✓ ✓ ✓ ✓ ✓ ✓ ✓ ✓ ✓  ✓ ✓ ✓ ✓ ✓ ✓ ✓ ✓ ✓  ✓ ✓ ✓ ✓ ✓ ✓ ✓ ✓ ✓  ✓ ✓ ✓ ✓ ✓ ✓ ✓ ✓ ✓  ✓ ✓ ✓ ✓ ✓ ✓ ✓ ✓ ✓  ✓ ✓ ✓ ✓ ✓ ✓ ✓ ✓ ✓  ✓ ✓ ✓ ✓ ✓ ✓ ✓ ✓ ✓  ✓ ✓ ✓ ✓ ✓ ✓ ✓ ✓ ✓ | ✓ ✓ ✓ ✓ ✓ ✓  – – – – – –  – – – – – –  – – – – – –  ✓ ✓ ✓ ✓ ✓ ✓  – – – – – –  ✓ ✓ ✓ – – –  – – – – – –  ✓ ✓ ✓ ✓ – ✓  ✓ ✓ ✓ ✓ – ✓  ✓ ✓ ✓ ✓ ✓ ✓  – – – – – –  ✓ ✓ ✓ ✓ ✓ ✓ | – – – – – –  – – – – – –  – – – – – –  – – – – – –  ✓ ✓ ✓ ✓ ✓ ✓  – – – – – –  – – – – – –  – – – – – –  – – – – – –  – – – – – –  ✓ ✓ ✓ ✓ ✓ ✓  – – – – – –  – – – – – – |
| Periplasmic Proteins | Q72VB8  Q72R59  Q72PY2  Q72V31 | gpmI  flab  glyA  purD | ✓ ✓ ✓ ✓ ✓ ✓ ✓ ✓ ✓  ✓ ✓ ✓ ✓ ✓ ✓ ✓ ✓ ✓  ✓ ✓ ✓ ✓ ✓ ✓ ✓ ✓ ✓  ✓ ✓ ✓ ✓ ✓ ✓ ✓ ✓ ✓ | ✓ ✓ ✓ ✓ ✓ ✓  ✓ ✓ ✓ ✓ ✓ ✓  ✓ ✓ ✓ ✓ ✓ ✓  – – – – – – | – – – – – –  ✓ ✓ ✓ ✓ ✓ ✓  ✓ ✓ ✓ ✓ ✓ ✓  – – – – – – |

*, *L. interrogans* serovar serogroup Icterohaemorrhagiae serovar Copenhageni (strain Fiocruz L1-130)

✓, conserved orthologs

–, not conserved

*ale., L. alexanderi; als., L. alstonii; bor., L. borgpetersenii; kir., L. kirschneri; kme., L. kmetyi; may., L. mayottensis; nog., L. noguchii; san., L. santarosai; wei., L. weilii; bro., L. broomii; fai., L. fainei; ina., L. inadai; lice., L. licerasiae; wolf., L. wolffii; ven., L. venezuelensis; bif., L. biflexa; mey., L. meyeri; ter., L. terpstrae; van., L. vanthielii; wolb., L. wolbachii; yan., L. yanagawae.*
